# Understanding the role of teamwork in recruitment to randomised controlled trials in surgical oncology-results from an exploratory study

**DOI:** 10.1186/1745-6215-16-S2-P82

**Published:** 2015-11-16

**Authors:** Sean Strong, Sangeetha Paramasivan, Caroline Wilson, Nicola Mills, Jenny Donovan, Jane Blazeby

**Affiliations:** 1 University of Bristol, Bristol, UK

## Background

Recruitment to trials is a process, involving a number of related stages completed by different healthcare professionals working as a team. In the UK, management decisions for patients with cancer are made within multi-disciplinary teams (MDTs) and therefore provide a forum where by effective teamwork and leadership may be important in determining successful trial recruitment. This study explored how MDT working may influence recruitment into randomised controlled trials (RCTs).

## Methods

Interviews were conducted with a purposeful sample of three cancer MDTs recruiting to a feasibility RCT comparing chemoradiotherapy with chemoradiotherapy and surgery for oesophageal squamous cell carcinoma. Interviews explored factors known to influence healthcare team effectiveness such as task design, team processes, team psycho-social traits and leadership style. Interviews were audio-recorded and analysed thematically. Sampling, data collection and analysis were undertaken iteratively and concurrently.

## Results

20 interviews with healthcare professional at three centres were performed (7 surgeons, 6 research nurses, 5 oncologists, and 3 specialist nurses. Group cohesiveness, including familiarity with team members, the duration a team had worked together and prior research experience were described as important traits of effective research teams. Shared study leadership positively influenced healthcare professionals’ willingness to participate. Competition over the number of patients randomised by individual healthcare professional and by different study sites further galvanised recruitment in high performing teams.

## Conclusions

This exploratory study has highlighted how team attributes such as group cohesiveness, shared leadership models and team psycho-social traits such as competiveness are important in high functioning research teams.

